# Severe nephrotic syndrome and early end-stage diabetic kidney disease in *ABCC8*-MODY12: A case report

**DOI:** 10.3389/fgene.2023.1132772

**Published:** 2023-03-15

**Authors:** Sophie H. Schmidt, Ursula Barnas, Christof Aigner, Peter Wolf, Nicolas Kozakowski, Renate Kain, Thomas Scherer, Alice Schmidt, Gere Sunder-Plassmann

**Affiliations:** ^1^ Department of Medicine III, Division of Nephrology and Dialysis, Medical University of Vienna, Vienna, Austria; ^2^ Medical School, Sigmund Freud University, Vienna, Austria; ^3^ Department of Medicine I, Clinic Landstraße, Vienna Healthcare Group, Vienna, Austria; ^4^ Department of Medicine III, Division of Endocrinology and Metabolism, Medical University of Vienna, Vienna, Austria; ^5^ Department of Pathology, Medical University of Vienna, Vienna, Austria

**Keywords:** *ABCC8* gene, MODY, diabetes mellitus, end-stage kidney disease, nodular glomerulosclerosis, sulfonylurea, case report

## Abstract

A 24-year-old man with diabetes mellitus presented with advanced kidney disease and severe proteinuria. Genetic testing revealed *ABCC8*-MODY12 (OMIM 600509), and a kidney biopsy showed nodular glomerulosclerosis. He commenced dialysis shortly thereafter, and glycemic control improved on treatment with a sulfonylurea. Diabetic end-stage kidney disease in patients with *ABCC8*-MODY12 has not been reported until now. Thus, our case highlights the risk for early-onset and severe diabetic kidney disease in patients with *ABCC8*-MODY12 and the importance of timely genetic diagnosis in unusual cases of diabetes to allow for proper treatment and prevention of late sequelae of diabetes.

## 1 Introduction

Maturity-onset diabetes of the young (MODY) is characterized by the onset of hyperglycemia at an early age. It is inherited in an autosomal-dominant pattern with pathogenic variants in at least 14 genes, accounting for less than 5% of the diabetes cases. Among those, *ABCC8*-MODY12 is a very rare form, and diabetic kidney disease in such patients has not been reported so far (Lin et al., 2020; Timmers et al., 2021).

## 2 Case description

**FIGURE 1:**
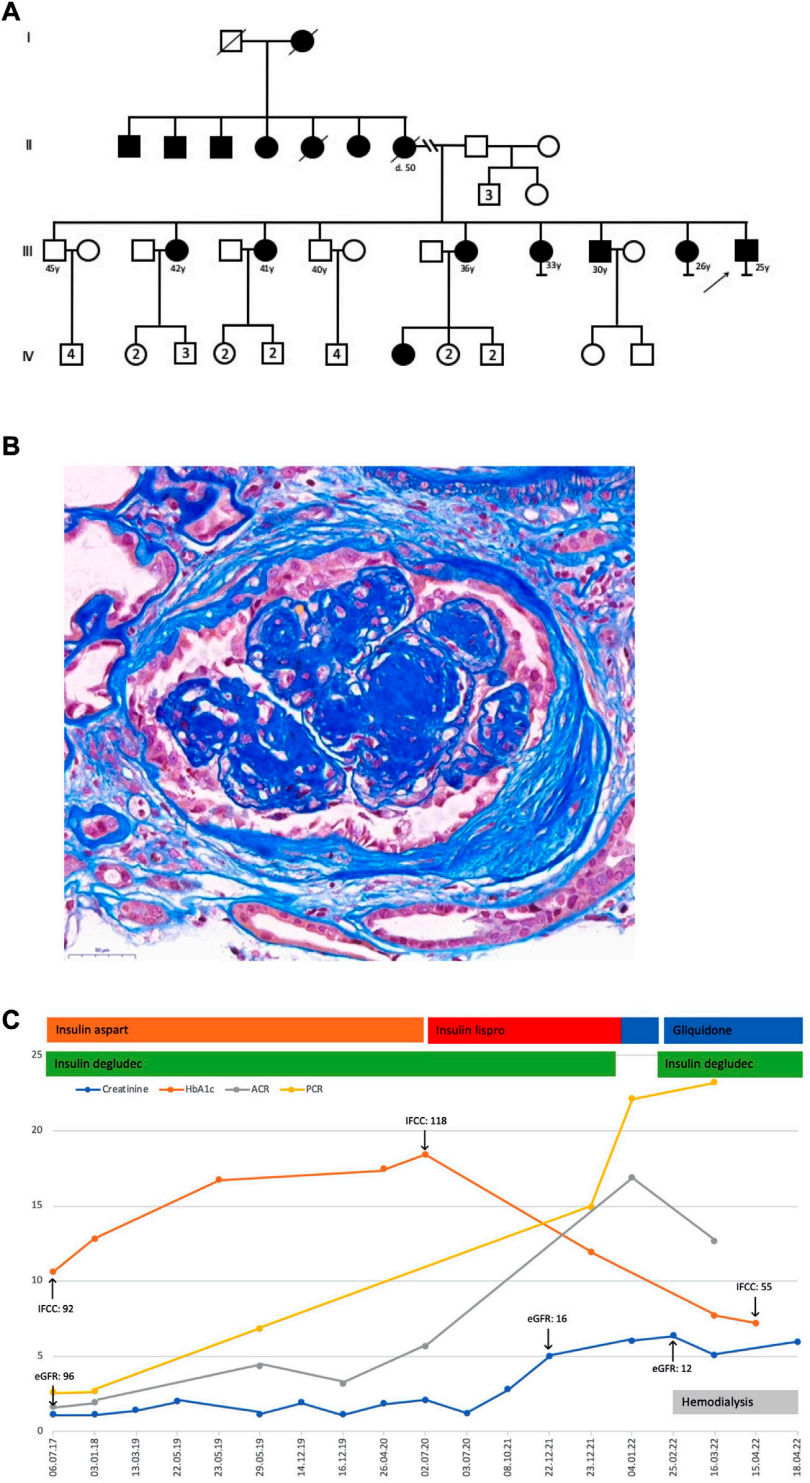
**(A)** Pedigree. **(B)** Kidney biopsy displayed 17 glomeruli, seven glomerular scars, and two arteries. The glomeruli had a clear and nodular mesangial matrix expansion, a focal and low-grade mesangial hypercellularity, and segmental hyaline lesions. Arteries demonstrated a discrete intimal expansion, whereas arterioles showed a major transmural hyalinosis with significant luminal narrowing. A diffuse and variable, often prominent, interstitial fibrosis with tubular atrophy, accompanied by a low-grade lymphomononuclear interstitial infiltrate and a slight tubular cell dystrophy with regenerative changes, was noticeable. The immunohistochemistry only showed IgM and C1q deposits in sclerosed glomerular portions. These findings were consistent with advanced diabetic nephropathy with nodular glomerulosclerosis, significant arteriolosclerosis, and major interstitial fibrosis with tubular atrophy. The electron microscopic examination of a partially sclerotic glomerulus confirmed glomerular changes compatible with diabetic damage, exhibiting pauci-cellular mesangial expansion, broadened capillary basal membranes due to an enlarged lamina densa, and some double contours. Podocyte structures showed flattened pedicels in the rare non-sclerotic segments. Additionally, some subendothelial electron-dense and often electron-lucent areas consistent with partially dissolving immune deposits suggested a concomitant and resolving immune complex glomerulonephritis. **(C)** Laboratory results and treatment details; creatinine, mg/dL; HbA1c, %; ACR, albumin-to-creatinine ratio, g/g; PCR, protein-to-creatinine ratio, g/g; eGFR, estimated glomerular filtration rate, mL/min per 1.73 m^2^; IFCC, HbA1c IFCC, mmol/mol.

In 2012, a Syrian male teen aged 15 years fled his home country because of war. Upon entering Turkey, he then reached Bulgaria. During the height of the Syrian migrant crisis in Europe, he arrived in Austria in 2015. The non-obese teen with a low average height of 155.5 cm was diagnosed with type 1 diabetes in the first 10 years of his life. His family history disclosed several relatives with a diagnosis of diabetes not characteristic of type 1 or 2, many of them living in Syria (the pedigree of this family is shown in Figure 1A). In Austria, the teen was reported with recurrent uncontrolled hyperglycemia and microvascular complications, including retinopathy, neuropathy, and osteomyelitis due to diabetic foot ulcers, which resulted in frequent hospital admissions. In 2021, the then 24-year-old Syrian migrant was conclusively diagnosed with *ABCC8*-MODY12, and a course of sulfonylurea was administered instead of insulin. It is worth noting that insulin therapy was eventually re-introduced. Next-generation sequencing and analysis of a MODY panel revealed a likely pathogenic missense variant in exon 10 of *ABCC8* (c.1616A>G; p. Tyr539Cys; heterozygous; ClinVar ID 35606) and ruled out relevant variants in other genes (*AKT2*, *APPL1*, *BLK*, *CEL*, *CELP*, *EIF2AK3*, *FOXP3*, *GATA6*, *GCK*, *GCKR*, *GLIS3*, *GLUD1*, *HADH*, *HNF1A*, *HFN1B*, *HNF4A*, *HYMAI*, *INS*, *INSR*, *KCNJ11*, *KLF11*, *NEUROD1*, *PAX4*, *PDX1*, *PLAGL1*, *PTF1A*, *SLC16A1*, *SLCA2*, *WFS1*, and *ZFP57*). Serum C-peptide and proinsulin were in the reference range, suggesting that endogenous insulin production was present and autoantibodies to glutamic acid decarboxylase (GAD), insulin (IAA), and protein tyrosine phosphatase (IA–2A) were undetectable. Furthermore, no diabetic ketoacidosis was reported.

Kidney function in this patient was not impaired upon arrival in Austria (CKD G1A3) but gradually declined thereafter. In parallel, worsening of albuminuria and proteinuria was recorded with albumin-to-creatinine and protein-to-creatinine ratios of up to 16.9 g/g and 22.1 g/g, respectively. In 2022, at the age of 25 years, a kidney biopsy showed severe nodular glomerulosclerosis (the kidney biopsy is shown in Figure 1B). In the present case, severe proteinuria did not respond to conventional medical therapy, and he commenced hemodialysis a few weeks after his kidney biopsy. Persistent severe nephrotic syndrome after initiation of renal replacement therapy necessitated medical nephrectomy with a high dose of angiotensin receptor blocker and non-steroidal anti-inflammatory drug therapy, which was, however, not successful. After the addition of gliquidone to insulin therapy, glucose levels were fairly well controlled, with an HbA1c of 55 mmol/mol. Notably, glycemic control was not achieved with insulin therapy alone and markedly improved after definitive genetic diagnosis and the addition of a sulfonylurea (Figure 1C **)**.

## 3 Discussion

Pathogenic or likely pathogenic variants in *ABCC8* are present in about 15% of individuals with neonatal diabetes and in some 2% of patients with MODY-monogenic diabetes (De Franco et al., 2015; Gaál et al., 2021; Rafique et al., 2021). With regard to diabetic kidney disease, microalbuminuria was indeed observed in some patients with *ABCC8*-MODY12. However, diabetic end-stage kidney disease in patients with *ABCC8*-MODY12 has not been reported until now. Thus, our case highlights 1) the potential risk for early-onset and severe diabetic kidney disease in patients with *ABCC8*-MODY12 and 2) the importance of timely molecular genetic diagnosis in unusual cases of diabetes (young age, positive family history of diabetes, and detectable endogenous insulin production with negative diabetes-associated antibodies) to allow for proper treatment, including SGLT2i medication, and prevention of late sequelae of diabetes (Ovsyannikova et al., 2016).

## 4 Patient consent

The patient provided written informed consent for the publication of this case report.

## Data Availability

The original contributions presented in the study are included in the article; further inquiries can be directed to the corresponding author.
